# An exploration of the association between premorbid weight status on patient and caregiver factors at pre and post-treatment among youth with anorexia nervosa/atypical anorexia nervosa

**DOI:** 10.1016/j.eatbeh.2023.101786

**Published:** 2023-07-23

**Authors:** Rachel Kramer, Rachel Radin, Sarah Forsberg, Andrea K. Garber, Erin E. Reilly, Lisa Hail, Kathryn M. Huryk, Jessica Keyser, Lindsey D. Bruett, Daniel Le Grange, Sasha Gorrell, Erin C. AccursO

**Affiliations:** a Department of Psychiatry and Behavioral Sciences, University of California, San Francisco, San Francisco, CA, USA; b Department of Pediatrics, University of California, San Francisco, San Francisco, CA, USA

**Keywords:** Atypical anorexia nervosa, Anorexia nervosa, Premorbid weight status, Caregivers, Treatment outcome

## Abstract

Patients with atypical anorexia nervosa (AAN) or anorexia nervosa (AN) with premorbid history of higher weight (PHW; median BMI ≥ 85th %ile) may report greater eating disorder (ED) pathology, anxiety, and depression, than patients with premorbid history of lower weight (PLW; mBMI <85th %ile). Less is known about caregiver attitudes or treatment outcome related to premorbid weight history. The current study examined associations between premorbid weight history and patient/caregiver factors at presentation, during treatment, and end of treatment among adolescents (*N* = 138) diagnosed with AN/AAN and their caregivers who received interdisciplinary ED treatment. The sample comprised adolescents with PHW (*n* = 58, 40.6 %) or PLW (*n* = 82, 59.4 %). Adolescents with PHW did not differ with regard to patient- or caregiver-reported ED symptoms, comorbid psychopathology, rates of treatment completion, and attainment of estimated body weight compared to PLW (*ps* > .05). Adolescents with PHW (vs. PLW) were more likely to be diagnosed with AAN (67.9 %, *p* < .001), identify as cisgender male (*p* < .001) and to have lost more weight prior to presentation (*p* < .001). Perceived caregiver burden was lower among adolescents with PHW vs. PLW (*p* < .001). Further research should expand on this preliminary study exploring associations between premorbid weight history on patient and caregiver factors at treatment presentation and conclusion to enhance the efficacy of evidence-based treatment across the weight-spectrum.

## 1. Introduction

Anorexia nervosa (AN) and atypical AN (AAN) are eating disorders (EDs) characterized by weight loss or failure to grow in accordance with expected trajectories, associated body-image disturbance, and functional impairment. Despite significant weight loss and symptom severity, individuals with AAN typically present “at or above normal weight” (American Psychiatric Association (APA), 2013). Despite historical evidence of treatment delays among those with AAN, research now indicates similar rates of medical admissions, medical risk, and a greater prevalence of AAN than AN among adolescents (Harrop et al., 2021; Sawyer et al., 2016; Whitelaw et al., 2014).

Yet, AN and AAN classifications do not take into account premorbid weight history (weight trajectory prior to ED development), meaning that some adolescents would have to lose significantly more body mass than others to meet criteria for AN (Garber et al., 2019; Whitelaw et al., 2014) which is concerning since the magnitude and rate of weight loss, rather than weight at initial treatment presentation, are stronger predictors of medical complications across all AN-type diagnoses (Garber et al., 2019; Sawyer et al., 2016). Total body mass loss (i.e., weight suppression, or the degree of weight an individual loses from their highest weight) and weight history may also be important to consider, given its relation to ED symptom severity (Jenkins et al., 2018). Adolescents with premorbid history of higher weight (PHW; BMI ≥ 85th % ile, labeled as having overweight/obesity by the United States Center for Disease Control [CDC] guidelines) endorse higher levels of ED psychopathology and body dissatisfaction (Matthews et al., 2021; Peebles et al., 2010; Swenne, 2016), depression and anxiety (Matthews et al., 20221; Monge et al., 2015; Shachar-Lavie et al., 2022), with overall similar psychiatric comorbidities, psychotropic medication use, and past mental health treatment (Matthews et al., 2022), compared to patients with AN or patients with premorbid lower weight (PLW; BMI < 85th %ile), regardless of weight suppression. Adolescents with PHW may also endorse greater body dissatisfaction (Calzo et al., 2012), weight stigma (Neumark-Sztainer, 2009; Pont et al., 2017), and weight-based teasing (Matthews et al., 2021), placing them at greater risk for ED (Haines & Neumark-Sztainer, 2006; Lie et al., 2019). These data suggest that PHW may be associated with a similar or more severe clinical profile (Matthews et al., 2021), particularly in light of well-documented weight stigma and misperceptions regarding the correlation between weight and health (Harrop et al., 2021; Neumark-Sztainer, 2009; Puhl & Heuer, 2009).

Less is known about how premorbid weight history impacts caregiver perception of ED symptoms or burden in the context of adolescent and young adult EDs, which is high throughout ED treatment (Kyriacou et al., 2008; Rhind et al., 2016; Zabala et al., 2009). Potential weight biases related to ED detection (Schaumberg et al., 2017), mixed messages received from medical providers (e.g., continued recommendations for weight loss despite significant ED behaviors and weight suppression), or even individual weight concerns among caregivers could predict different perceptions of ED severity. Caregivers may experience different perceptions of burden related to their child’s weight history (e.g., systemic weight stigma) or internalized weight bias (Rogers et al., 2019), which may also impact caregiver focus on weight restoration efforts during Family Based Therapy (FBT) (Kimber et al., 2019).

Taken together, improved understanding from both the patient and caregiver perspective of the impact of premorbid weight status on ED treatment is critically important. Some research suggests adolescents with AAN gain less weight during treatment (Hughes et al., 2017; Lebow et al., 2019), but existing research has methodological limitations (e.g., case series: Hughes et al., 2017; data collected prior to treatment completion: Lebow et al., 2019). Research exploring weight suppression has also yielded mixed results and has been conducted more frequently with patients with AN (Jenkins et al., 2018). No studies were identified that have explored associations between premorbid weight status and treatment outcome. Research on how premorbid weight history impacts treatment initiation, treatment recommendations, and outcome (including achievement of estimated goal weight) is warranted to appreciate factors affecting treatment beyond diagnostic status at presentation.

The current study had three aims. The first aim was to replicate previous research examining differences related to premorbid weight status among adolescents with AN/AAN at treatment presentation. The second aim was to explore caregiver perceptions of ED severity and burden relative to premorbid weight status. The third aim was to examine whether premorbid weight status contributed to meaningful differences in the proportion of adolescents initiating outpatient treatment in an academic medical center after initial assessment, treatment type recommended, need for higher level of care, number of sessions, length of treatment, and weight outcomes (percent of estimated body weight determined by historical growth charts). We hypothesized adolescents with PHW would endorse greater ED symptom severity, depression, and anxiety than PLW; there were no a priori hypotheses for Aims 2 or 3 given lack of prior research.

## 2. Methods

### 2.1. Participants and procedure

This study comprised 138 adolescents (aged 10–18) diagnosed with AN/AAN who presented to an interdisciplinary ED treatment program in the United States from September 2015 to March 2020. As part of routine care, patients and caregivers completed questionnaires prior to initial intake and agreed to have their medical charts reviewed until discharge from therapy with a psychologist or social worker from our team. ED and comorbid diagnoses were conferred based on psychological evaluation with adolescents, their caregivers, and results from the Eating Disorder Assessment-5 (Sysko et al., 2015), Eating Disorders Examination interview (Fairburn & Beglin, 1994), and Mini International Neuropsychiatric Interview for Children and Adolescents (Sheehan et al., 2010). Caregivers and adolescents provided informed assent/consent to participate in this study, approved by the site’s Institutional Review Board. Data from one caregiver (self-designated as the “primary” caregiver) per patient was included in this study for parsimony.

### 2.2. Premorbid weight history

Premorbid weight status was determined from historical growth records and identifying the highest BMI percentile trend based on caregiver report and review of medical charts while accounting for any outliers. Patients were then classified into premorbid weight-status groups based on their highest BMI percentile and guidelines from the CDC (2017). Combining across diagnostic category, adolescents with AN/AAN were either classified as having premorbid lower weight (PLW, highest BMI < 85th percentile) or premorbid history of “higher weight” (PHW; highest BMI ≥ 85th percentile, labeled “overweight” by the CDC).

### 2.3. Medical chart review

The following variables were extracted from electronic health records: age, gender identity, race, ethnicity, medication prescribed (yes/no), psychiatric comorbidity (yes/no and diagnosis), illness duration (months), number of sessions attended, length (in months) of treatment (from first session to discharge), participation in other levels of care, reasons for not continuing in care, highest premorbid BMI percentile and percent of median BMI (%mBMI), baseline BMI percentile and %mBMI. Rate of weight loss was determined by dividing %mBMI loss (%mBMI at highest weight to baseline) by illness duration (months) (Garber et al., 2019) and all %mBMI were based on age-and-sex based norms (CDC, 2017) Weight suppression was calculated by subtracting baseline % mBMI from the highest %mBMI (Accurso et al., 2023; Witt et al., 2014). Expected body weight (EBW) and percent of EBW (%EBW) at baseline were determined and entered into health records by dietitians via growth chart review. Percent of EBW at 3 months and end-of-treatment (EOT) were calculated by subtracting EBW from weight at these time points, dividing by EBW, and multiplying by 100.

### 2.4. Self-report questionnaires

#### 2.4.1. Patient measures

##### 2.4.1.1. Eating disorder symptoms.

ED symptoms were assessed via self-report using the Eating Disorder Examination-Questionnaire (EDE-Q; Fairburn & Beglin, 1994). The EDE-Q is a widely used 28-item measure that assesses ED pathology, with good psychometric properties among girls, boys, and gender-diverse populations (Mond et al., 2006; Peterson et al., 2020; Smith et al., 2017). The EDE-Q global score was used for this study with excellent internal consistency (α = .97).

##### 2.4.1.2. Depressive symptoms.

Depressive symptoms were assessed using the 12-item Child Depression Inventory-2 Short form (CDI-2SF; Kovacs et al., 2010). The CDI-2 demonstrates adequate screening abilities, validity, and reliability among youth (Houghton et al., 2022). Internal consistency was adequate (α = .86).

##### 2.4.1.3. Anxiety symptoms.

Anxiety was assessed using the Multidimensional Anxiety Scale for Children – Short form (March 1997), a 10-item self-report questionnaire with considerable validity and reliability (March et al., 1997). Internal consistency was acceptable (α = .77).

#### 2.4.2. Caregiver measures

##### 2.4.2.1. Parent report of adolescent ED symptoms.

The Parent Eating Disorder Examination-Questionnaire (PEDE-Q; Loeb, 2008) assessed caregiver perception of their child’s ED severity. Internal consistency for the global PEDE-Q score used for this study was excellent (α = .92).

##### 2.4.2.2. Caregiver burden.

The Caregiver Strain Questionnaire-Revised short form (CSQ; Brannan et al., 2012) is a 7-item Likert-based scale with high scores indicating caregiver strain. The CSQ demonstrates good reliability and validity (Brannan et al., 2012). Internal consistency was acceptable (α = .85).

### 2.5. Data analytic plan

Descriptive statistics were calculated to describe both premorbid weight status groups. Data analysis was performed using SPSS version 27; to account for multiple comparisons, significance was set at *p* < .044 for all analyses.

#### 2.5.1. Aim 1

Chi-square, Fisher’s Exact, or Fisher-Freeman-Halton Exact tests (for cell counts <5) were used to explore associations of premorbid weight status on presenting characteristics including gender identity (cisgender or transgender male or female), ED diagnosis (AN/AAN), race, ethnicity, psychotropic medication use, and co-occurring psychiatric diagnoses.

ANOVAs were used to explore group differences related to age, illness duration, EBW, %EBW at baseline, baseline and premorbid (highest weight) BMI percentile and %mBMI, and weight suppression. ANCOVA was used to explore patient-level group differences for Aim 1 (EDE-Q global, MASC, CDI scores) while controlling for weight suppression. Cisgender boys reported lower EDE-Q, *F*(1, 53) = 11.62, *p* = .001, and CDI *F*(1, 52) = 13.11, *p* < .001, scores than cisgender girls (See Table 2), thus we adjusted for gender identity for ANCOVAs examining EDE-Q and CDI scores.

#### 2.5.2. Aim 2

ANCOVAs, adjusting for weight suppression, examined caregiver perception of ED severity (PEDE-Q), and caregiver burden (CSQ).

#### 2.5.3. Aim 3

Chi-square and Fisher’s Exact tests were used to compare effects of premorbid weight status (group) on the proportion of patients assessed who initiated outpatient treatment, which treatment was recommended (among treatment completers), and need for a higher level of care. Given our modest sample, Kruskal-Wallis tests were used to assess differences in treatment length and number of treatment sessions. A repeated measures ANCOVA (controlling for weight suppression and treatment length and using multivariate F and *p*-values) was conducted with posthoc tests exploring differences between patients with AN/AAN with PHW vs. PLW in %EBW change from baseline to 3-months and EOT.

## 3. Results

Descriptive results are available in Table 1. The sample included outpatients diagnosed with AN (*n* = 73, 52.9 %) or AAN (*n* = 65, 47.1 %) between 10 and 18 years of age, *M*(*SD*) = 15.20 (13.69). The majority identified as Non-Hispanic (85.0 %), White (74.2 %), and cisgender female (80.0 %). Fifty-eight (40.6 %) were classified with PHW and 82 with PLW (59.4 %). Among caregivers, 106 (80.3 %) were mothers, 25 (18.9 %) were fathers, and 1 (0.8 %) was a grandmother.

### 3.1. Aim 1

Gender identity (Fisher’s exact, *p* < .001), diagnosis at baseline (*χ*^*2*^(1) = 16.30, *p* < .001), prior psychotropic medication use *χ*^*2*^(1) = 8.54, *p* = .003), and ethnicity (*χ*^*2*^(1) = 7.53, *p* = .006) were associated with premorbid weight status. A larger portion of adolescents with PHW (vs. PLW) were cisgender male vs. female, diagnosed with AAN vs. AN, and were Hispanic vs. Non-Hispanic. A smaller portion of adolescents with PHW were using psychotropic medications at baseline. Patients with PHW had significantly greater premorbid BMI percentile and % mBMI, baseline BMI percentile and %mBMI, weight suppression, and higher EBW goal weights than adolescents with PLW (Table 2). Groups did not significantly differ on illness duration, %EBW at baseline, ED symptoms, depression, or anxiety, or previous mental health diagnosis (Tables 1 and 2).

### 3.2. Aim 2

Premorbid weight status was not significantly related to caregiver report of ED symptoms (Table 2). However, caregiver burden was associated with premorbid weight status such that caregivers of adolescents with PHW reported lower perceived burden compared to caregivers of adolescents with PLW (*p* = .006).

### 3.3. Aim 3

**Fig. 1.**
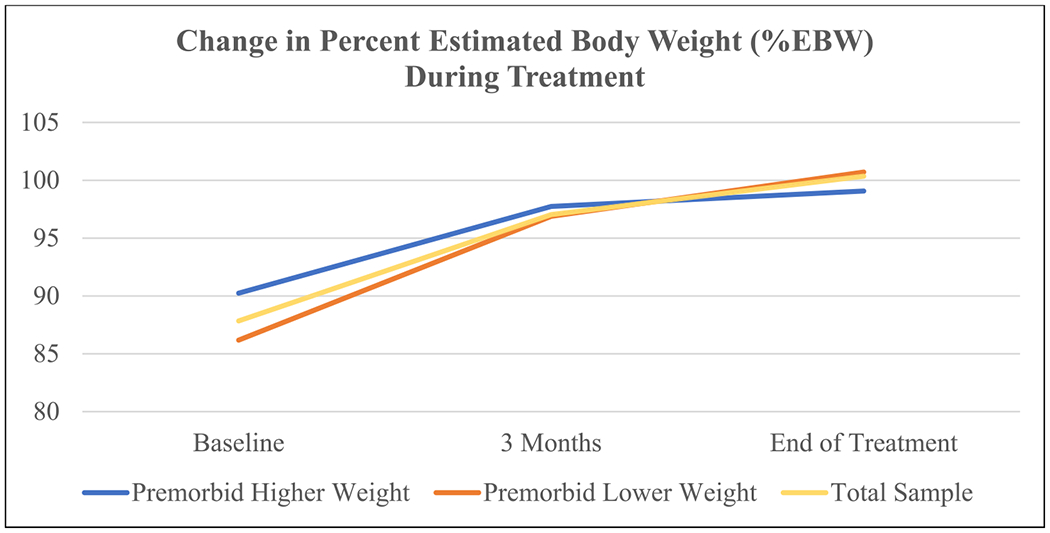
Change in %EBW over the course of treatment among groups.

Eighty-five of 131 patients assessed at baseline (65 %) initiated outpatient treatment; these rates were similar regardless of premorbid weight status, *χ*^*2*^(1) = 0.27, *p* = .604. Adolescents who did not initiate treatment were excluded from Aim 3 analyses, with no differences related to premorbid weight status (Fisher’s *p* = .828). These patients were referred to higher levels of care (*n* = 6), sought care with community or previously established providers (*n* = 16), did not follow postassessment (*n* = 12), were unable to secure insurance coverage (*n* = 2), moved away (*n* = 2), or shared their child was improving eating on their own (*n* = 3). Recommended treatment approach was not associated with premorbid weight status (Fisher’s exact, *p* = .644); almost all treatment recommendations (94 %) were for Family-Based Treatment (FBT; Lock & Le Grange, 2013). There were no significant differences in treatment length or number of treatment sessions related to premorbid weight status. Need for engagement in higher levels of care (e.g., intensive family therapy, residential, partial hospitalization, or medical inpatient) was also not different between adolescents with PWH vs. PWL, *χ*^*2*^(1) =0.06, *p* = 1.00. Adolescents with PHW had higher EBWs compared to adolescents with PLW (*p* = .003). A significant interaction effect between premorbid weight status and time *F*(1, 1.73) = 4.38, *p* = .022, $\eta_{\text{p}}^{2}$ = 0.13, suggested at baseline, adolescents with PHW were at a higher % EBW compared to those with PLW. However, premorbid weight status was not associated with %EBW at 3 months or EOT and %EBW improved for both groups at all time points (Fig. 1 and Table 2).

## 4. Discussion

This study aimed to replicate research exploring associations between premorbid weight status (adolescents with either PHW or PLW) on characteristics of adolescents diagnosed with AN/AAN, caregiver perceptions of ED severity, and caregiver burden. Finally, because limited work has examined clinical differences related to premorbid weight status in the context of ED treatment, this study aimed to explore characteristics of outpatient treatment assignment and treatment course related to premorbid weight status.

PHW was present in adolescents with both AN (about one third) and AAN (about two-thirds), similar to previous research (Lebow et al., 2015; Matthews et al., 2022, 2021; Sawyer et al., 2016). Our data demonstrated that adolescents with PHW had higher BMI percentiles and greater weight suppression at treatment initiation, despite no differences in illness duration. This finding may suggest improved detection of ED also observed more recently in our clinic and others (Garber et al., 2019; Matthews et al., 2022, 2021). It is difficult to speculate why cisgender males were more likely to have PHW, but Calzo et al. (2012) have reported that cisgender boys with BMI percentiles ≥75th percentile endorse the highest levels of body dissatisfaction compared to cisgender boys with lower BMI percentiles, perhaps increasing risk for ED development within this demographic.

Further, a greater portion of Hispanic youth had PHW. While available measurements cannot speak directly to the processes contributing to observed differences, data supports increased risk for EDs among Latinx youth, particularly those with higher weights (Hernández et al., 2022; Rodgers, Peterson, et al., 2017; Rodgers, Watts, et al., 2017), and suggests that intersecting identities associated with stigma and minority stress processes may contribute to increased clinical ED presentations in this group (Burke et al., 2020). However, despite this increased risk, research also indicates under-detection of ED among minority youth (Schaumberg et al., 2017) and lack of appropriateness of BMI metrics in racial and ethnic minority samples (Adab et al., 2018).

Our findings also suggest that ED symptoms, anxiety, and depression did not differ by premorbid weight status, reinforcing the severity of ED behaviors regardless of weight history, and in contrast to previous findings suggesting that adolescents with AAN or PHW report elevated distress (Hughes et al., 2017; Matthews et al., 2021; Sawyer et al., 2016). We may not have detected differences between groups on anxiety, depression, or ED symptoms due to general ceiling effects (all groups being distressed) or similar illness durations which is associated with ED severity. This is one of the first studies to explore associations between premorbid weight status and caregiver perception of ED severity and caregiver burden. Regardless of their child’s weight history, caregivers observed significant ED symptoms. This is potentially congruent with research demonstrating commensurate medical risks across AN-diagnostic type (Garber et al., 2019; Whitelaw et al., 2018) which we noted. Similar portions of PHW and PLW were medically admitted and referred to higher levels of care. Further, most patients were referred to the specialized ED program through PCPs and thus had similar referral processes and medical advice on medical concerns related to ED. Additionally, caregivers were most likely treatment initiators suggesting caregivers regardless of premorbid weight history noted concerning ED symptoms among their children. Lastly, the lack of significant group differences in adolescent endorsement of ED severity may also correspond to equivalent reports of ED symptoms among caregivers. However, caregivers endorsed lower caregiver burden when their child had PHW vs. PLW. Since subjective caregiver perceptions of ED severity are associated with burden (Matthews et al., 2018), caregivers of patients with PHW may have felt less anxious about their child’s health at presentation given their historic weight status or due to internalized weight bias. This aligns with societal beliefs around associations between weight and health, and reported clinician observations (Dimitropoulos et al., 2019; Kimber et al., 2019).

In terms of treatment initiation and outcome, a large majority of patients were recommended and completed FBT (94 %). Treatment length, intensity level, and session frequency were similar among weight-history groups. While adolescents with PHW had higher EBWs compared to those with PLW, most adolescents achieved weight restoration (acheiving EBW based on their growth curve) by EOT. This finding is promising since previous studies have defined recovery as reaching the median or 50th BMI%tile (Silén et al., 2015), which may not be an appropriate definition of physical recovery or indication of medical stability (Garber et al., 2019).

### 4.1. Limitations and suggestions for future directions

Future research could improve on the current study by increasing sample size to enhance power, as well as utilizing a more diverse sample of adolescents (e.g., inclusive of adolescents with public insurance who are more racially and ethnically diverse and not currently able to seek care at the present academic medical center). Further, we utilized methods such as Matthews et al. (2021) to determine premorbid weight status, while other studies have taken the median of premorbid BMI percentiles which could impact the ability to compare our results with other samples. Since weight status was dichotomized, there is little understanding of how weight fluctuation might impact factors assessed in this study, and we are still relying on historical labels which could perpetuate weight bias (Meadows & Daní;elsdóttir, 2016). The study was conducted at an interdisciplinary academic medical center which aims to address weight bias by setting EBW based on historical growth charts (versus population norms) and the clinic strives to utilize a Health At Every Size Approach (Burgard, 2009). Our program accepts patients not based on weight at presentation, but by presenting medical and psychological concerns and weight loss/lack of growth (Garber, 2018; Peebles & Sieke, 2019). Another limitation was that we did not measure weight bias, which could have helped expand understanding of potential moderators of results (e.g. differences in caregiver burden). Future research should examine how weight bias is related to premorbid weight status and outcomes.

Further research is needed to explore treatment outcome across more settings and among those who are not treatment seeking to generalize findings more broadly since caregivers receiving support may experience less burden compared to families not receiving care. Lastly, adolescents’ or caregivers’ experiences of weight bias/stigma, weight-based teasing, or encouragement for weight loss by medical providers was not assessed and precluded us from evaluating any mediating factors which could explain hypothesized group differences and future research should expand on this.

### 4.2. Conclusion

In sum, our findings indicate regardless of pre-morbid weight history, adolescents with AN/AAN and their caregivers report no differences in anxiety, depression, and ED symptoms. However, perceived caregiver burden was lower among caregivers of adolescents with AN/AAN and PHW. This finding may coincide with societal views that ED severity relates to weight loss, at least at treatment onset. Nevertheless, treatment proceeded similarly regardless of weight status, with most adolescents achieving weight restoration to their historical growth curve by end of treatment. This study contributes to the literature by providing a more nuanced evaluation of how weight history impacts patients and caregivers beyond weight at diagnosis and suggests caregivers may experience differences in burden related to their child’s weight history.

## Figures and Tables

**Table 1:** Demographic characteristics of adolescents with AN/AAN by premorbid weight status.

	Premorbid higher weight *n* = 56 (40.6%)	Premorbid lower weight *n* = 82 (59.4 %)	Total *n* = 138	Stat and *p*-value	Effect size
**ED Diagnosis**	*n* (%)	*n* (%)	*n* (%)	*χ*^*2*^(1) = 16.30, p <. 001	Φ = .34
Anorexia nervosa	18 (32.1)	55 (67.1)	73 (52.9)		
Atypical anorexia nervosa	38 (67.9)	27 (32.9)	65 (47.1)		
**Gender**				Fisher’s Exact, *p* = <.001	Cramer’s V = .35
Cisgender female	22 (22.9)	74 (90.2)	96 (80.0)		
Cisgender male	12 (70.6)	5 (6.1)	17 (14.2)		
Transgender female	0 (0.0)	3 (3.7)	3 (2.5)		
Transgender male	4 (10.5)	0 (0.0)	4 (3.3)		
**Race**				FFHE = 2.21, *p* =.756	Cramer’s V = .13
American Indian/Alaska Native	3 (5.6)	2 (2.4)	5 (3.7)		
Asian	4 (7.4)	6 (7.3)	10 (7.4)		
Black or African American	0 (0.0)	1 (1.2)	1 (0.7)		
Bi/Multiracial	5 (9.3)	13 (15.9)	18 (13.2)		
White	42 (77.8)	60 (73.2)	102 (75.0)		
**Ethnicity**				*χ^2^*(1) = 7.53, *p* = .006	Φ = .23
Hispanic	15 (27.8)	8 (9.8)	23 (16.9)		
Non-Hispanic	39 (72.2)	74 (90.2)	113 (83.1)		
**Taking psychotropic medication**				*χ*^*2*^(1) = 8.54, *p* = .003	Φ = .25
Yes	46 (82.1)	48 (58.5)	94 (68.1)		
No	10 (17.9)	34 (41.5)	44 (31.9)		
**Psychiatric comorbidity**				*χ*^*2*^(1) = 0.82, *p* = .366	Φ = .08
Yes	37 (68.5)	59 (75.6)	96 (72.7)		
No	17 (31.5)	19 (24.4)	36 (27.3)		
**Comorbid Diagnoses**					
DSM-5 depressive disorders	19 (35.2)	31 (39.7)	50 (37.9)		
DSM-5 bipolar disorders	0 (0.0)	1 (1.3)	1 (0.8)		
DSM-5 anxiety disorders	14 (25.9)	20 (25.6)	34 (25.8)		
DSM-5 OCD	3 (5.6)	1 (1.3)	4 (3.0)		
ADHD	0 (0.0)	2 (2.6)	2 (1.5)		
PTSD	0 (0.0)	1 (1.3)	1 (0.8)		
ODD	0 (0,0)	1 (1.3)	1 (0.8)		
Pervasive developmental disorder	1 (1.9)	1 (1.3)	2 (1.5)		
**Higher level of care during outpatient treatment** ^a^				*χ^2^*(1) =0.06, *p* = 1.00	Φ = .02
No	24 (77.4)	38 (77.6)	62 (77.5)		
Any higher level of care	7 (24.1%)	11 (26.8%)	18 (21.2)		
Medical admission	6 (19.4)	4 (8.2)	10 (12.5)		
Partial hospitalization/residential	0 (0.0)	5 (10.2)	5 (6.3)		
Intensive family therapy	1 (3.2)	2 (4.1)	3 (3.8)		

Note: AN = anorexia nervosa; AAN = atypical anorexia nervosa; OCD = obsessive-compulsive disorder; ADHD = attention-deficit/hyperactivity disorder; PTSD = post-traumatic stress disorder; ODD = oppositional defiant disorder, FFHE = Fisher-Freeman-Halton Exact tests.

a Among treatment completing adolescents (*n* = 85).

**Table 2:** Clinical characteristics of adolescents with AN/AAN and their caregivers at treatment presentation and treatment outcome.

	Premorbid higher weight *M* (*SD*)	Premorbid lower weight *M* (*SD*)	Total *M* (*SD*)	*F*	*P*	$\eta_{\text{p}}^{2}$
Age	15.01 (1.74)	15.34 (1.73)	15.20 (1.74)	1.20	.275	.01
Illness duration	15.50 (12.85)	13.43 (14.24)	14.26 (13.69)	0.74	.390	.01
**Weight-related variables**						
Premorbid BMI percentile	94.08 (7.32)	53.56 (22.08)	67.07 (26.63)	130.44	< .001	.43
Premorbid %mBMI	135.62 (20.64)	103.35 (15.72)	114.19 (23.20)	92.46	< .001	.51
Baseline BMI percentile	49.76 (27.69)	24.30 (21.60)	35.01 (26.93)	33.28	< .001	.19
Baseline %mBMI	103.46 (15.72)	90.91 (11.35)	95.89 (14.57)	29.21	< .001	.18
% EBW	87.37 (8.42)	87.14 (7.70)	87.23 (7.96)	0.02	.876	<.01
Rate of weight loss	4.58 (4.66)	4.95 (6.48)	4.82 (5.91)	0.01	.751	.001
Weight suppression	27.81 (20.01)	12.27 (14.97)	17.50 (18.30)	23.23	< .001	.15
**Self-report questionnaires (patient or caregiver)**						
Patient ED severity^a,c^	2.46 (1.72)	3.33 (1.69)	2.96 (1.75)	0.29	.590	.004
Patient depression^b,c^	70.26 (16.57)	75.40 (15.30)	73.14 (16.00)	0.37	.545	.003
Patient anxiety^c^	53.91 (2.02)	57.73 (1.77)	57.33 (13.61)	2.21	.140	.02
Caregiver reported ED severity^c^	3.30 (1.21)	3.01 (1.47)	3.11 (1.39)	0.32	.574	.004
Caregiver strain^c^	6.64 (1.71)	7.39 (1.56)	7.14 (1.64)	7.91	.006	.07
**Treatment variables**						
Treatment length (months)^d,g^	8.30 (7.68)	8.80 (7.02)	8.60 (7.24)	0.54	.461	.04
# of therapy sessions^d,g^	20.84 (16.38)	22.27 (16.57)	21.68 (16.40)	0.52	.472	.03
EBW (in lbs)	136.04 (16.90)	118.78 (16.89)	124.64 (18.65)	9.74	.003	.26
%EBW at baseline^c^	90.04 (8.01)	86.18 (7.27)	87.84 (7.21)	7.09	.010	.10
%EBW at 3 months^c^	97.74 (6.20)	96.88 (6.40)	97.03 (6.15)	0.11	.741	.002
%EBW at EOT^c,e^	99.07 (5.70)	100.71 (7.73)	100.35 (7.69)	1.54	.228	.03
Change in %EBW from baseline to three months^f^			*M*difference = 8.33		< .001	.13
Change in %EBW from three months to EOT^f^			*M*difference = 3.88		.003	.13
Change in %EBW from baseline to EOT^f^			*M*difference = 11.62		< .001	.17

a Controlling for gender, cisgender males (*M* = 1.87, *SD* = 1.52), cisgender females (*M* = 3.56, *SD* = 3.56),

b Controlling for gender, cisgender males (*M* = 62.62, *SD* = 18.05), cisgender females (78.68, *SD* = 12.44),

c Controlling for weight suppression,

d Kruskal-Wallis was used,

e Controlling for treatment length,

f ANCOV A within-subjects changes in %EBW (from treatment completing sample (n = 85),

g ε^2^ (Epsilon Squared was used for effect size).

Note: AN = anorexia nervosa; AAN = atypical anorexia nervosa; for the purposes of these data, diagnostic groups are combined. ED = eating disorder, EBW = Estimated Body Weight, %EBW = percent Estimated Body Weight, EOT = end of treatment.

**Weight Suppression** = difference between highest mBMI percentile to current mBMI percentile (age-and-sex matched).

**Measures:** Patient ED Symptoms (Eating Disorder Examination – Questionnaire), depression (Children’s Depression Inventory), anxiety (Multidimensional Anxiety Scale for Children-10), Caregiver ED severity (Parental Eating Disorder Examination – Questionnaire), Caregiver Family Functioning (Functional Assessment Device), Caregiver Strain (Caregiver Strain Questionnaire).

## Data Availability

The data that has been used is confidential.
